# Factors Associated With the Acceleration of Patient Enrollment in Clinical Studies: A Cross-Sectional Study

**DOI:** 10.3389/fphar.2021.753067

**Published:** 2021-10-27

**Authors:** Rieko Ueda, Yuji Nishizaki, Shuko Nojiri, Hiroshi Iwata, Katsumi Miyauchi, Kotone Matsuyama, Shoji Sanada, Tohru Minamino, Hiroyuki Daida

**Affiliations:** ^1^ Department of Cardiovascular Biology and Medicine, Juntendo University School of Medicine, Tokyo, Japan; ^2^ Medical Technology Innovation Center, Juntendo University, Tokyo, Japan; ^3^ Clinical Research and Trial Center, Juntendo Hospital, Tokyo, Japan; ^4^ Center for Strategic Research Initiative, Nippon Medical School, Tokyo, Japan; ^5^ Department of Health Policy and Management, Nippon Medical School, Tokyo, Japan; ^6^ Department of Medical Innovation, Osaka University Hospital, Osaka, Japan; ^7^ Center for Clinical Research and Innovation, Osaka City University Hospital, Osaka, Japan; ^8^ Faculty of Health Science, Juntendo University, Tokyo, Japan

**Keywords:** clinical research, clinical research coordinator, clinical research support, Japan, patient enrollment, site visit

## Abstract

Under-recruitment in clinical trials is an issue worldwide. If the number of patients enrolled is lower than expected, based on the required sample size, then the reliability of the study results and their validation tend to be impaired. The current study therefore evaluated factors associated with accelerating patient enrollment using data from an ongoing multicenter prospective cohort study. The researchers encouraged research institutions to accelerate patient enrollment *via* e-mail, newsletters, telephone calls, and site visits. We analyzed the relationship between several potential factors associated with acceleration of patient enrollment including site visits and patient enrollment in a real clinical study. Data were collected from 106 research institutions that participated in a multicenter prospective cohort study. Results showed that the following parameters differed in terms of patient enrollment and non-enrollment: urban area (47.2 vs. 67.6%, *p* = 0.04), clinical research coordinator (CRC) participation in data input to electronic data capture (EDC) (41.7 vs. 11.8%, *p* < 0.01), and site visit (38.9 vs. 11.8%, *p* < 0.01). A multivariate analysis revealed that patient enrollment was significantly associated with urban area (odds ratio [OR] 0.33, 95% confidence interval [CI] 0.12–0.86, *p* = 0.02), CRC participation in data input to EDC (OR 5.02; 95% CI 1.49–16.8; *p* < 0.01), and site visit (OR 4.54, 95% CI 1.31–15.7, *p* = 0.01). In conclusion, site visits and CRC participation in data input to EDC had a significant effect on patient enrollment promotion. Moreover, hospitals in rural areas were more effective in promoting patient enrollment than those in urban areas.

## Introduction

Completing patient enrollment within the enrollment period is vital for successful clinical research. Due to research ethics, target sample sizes are calculated in advance. If the number of enrollees is lower than the expected sample size, then the study results will not only be less reliable, the study will also be less valid and the hypothesis cannot be sufficiently validated.

A variety of achievement rates for patient enrollment in multicenter trials has been reported by the STEPS study. According to an epidemiological review of a cohort of trials funded by the Medical Research Council (MRC) and National Health Service (NHS) Health Technology Assessment (HTA) program, only 31% of 122 clinical trials achieved the required number of participants, with 45.1% achieving <80% target sample size (Campbell et al., 2007). Kitterman et al. examined all clinical studies receiving institutional review board (IRB) review from FY2006—FY2009 at Oregon Health and Science University (OHSU) for recruitment performance. The results of the survey showed that a total of 837 clinical studies were terminated during the study period, 260 (31.1%) of which were low-enrolling (Kitterman et al., 2011). To identify barriers to patient recruitment in clinical trials, Stein et al. conducted focus groups and key informant interviews with investigators, coordinators, and other stakeholders in clinical and translational research. They reported that the main barrier was a lack of support from clinical staff. Clinical practices are often utilized as the primary location for patient recruitment in clinical trials. However, the ability to recruit often relies on cooperation from clinicians and support staff. The focus group participants identified a lack of support from clinical staff as a barrier to recruitment. They felt that their studies were often seen as “extra work” or “intrusive” to the clinic’s workflow (Stein et al., 2015). A review article by Sully et al. found that publicly funded trials in the UK struggled to recruit their target sample size, and both time and financial extensions were often requested. Specifically, over half (55%) of the trials recruited the originally specified target sample size, and over three-quarters (78%) recruited 80% of their target. Strategies to cope with such challenges should be more widely applied. Based on the findings of Sully et al., it is recommended that, where possible, studies are planned with 90% power (Sully et al., 2013).

The Juntendo Clinical Research and Trial Center (JCRTC) fully supported the multicenter cohort study “Real World Anticoagulation and Antiplatelet Practice in Patients with Acute Coronary Syndrome Complicated with Atrial Fibrillation” (STAR-ACS) (University Hospital medical information network (UMIN), 2017), an ongoing, nationwide prospective cohort study involving 144 institutions (7.6% in Hokkaido, 2.8% in Tohoku, 36.1% in Kanto, 13.2% in Chubu, 17.4% in Kinki, 4.9% in Chugoku, 4.2% in Shikoku, and 13.9% in Kyushu). STAR-ACS aims to assess the real-world trends of acute coronary syndrome (ACS) complicated by atrial fibrillation and its associated bleeding and thrombotic events. The study enrolled patients with this condition who underwent coronary intervention during hospitalization and were treated with warfarin or direct oral anticoagulants at discharge. The study period is April 2016 to December 2021, and the target sample size is 460. The primary study endpoints are all-cause death, bleeding, and thrombotic events. The STAR-ACS study is a pilot observational study initiated primarily to investigate the incidence of bleeding and thrombotic events, drug usage, and drug usage duration. Therefore, the target number of cases was determined in consideration of feasibility. The study was registered under the University Hospital Medical Information Network (UMIN ID: 000027356) (University Hospital medical information network (UMIN), 2017). Patient enrollment started in April 2017, and 460 cases were registered by July 2019. Several strategies, including e-mail, newsletters, and telephone calls, were used to accelerate patient enrollment. However, the study experienced delayed patient enrollment at the beginning of the registration (April 2017–February 2018). The research centers were therefore requested to accelerate patient enrollment *via* site visits.

The current study aimed to investigate factors associated with accelerating patient enrollment using data from an ongoing multicenter prospective study.

## Materials and Methods

### Study Design

This study evaluated factors associated with accelerating patient enrollment using STAR-ACS data. We analyzed information from 106 research centers that registered no patients by February 2018, which was the start of the site visits. This study focused on the relationship between patient enrollment and site visits, among other factors.

### Site Visits

To facilitate clinical research, collaboration between the research secretariat, researchers, and support staff in each center is important. The research secretariat should understand the study’s outline and purpose, assess its progress in all centers in real time, and share information with researchers and support staff. However, there is concern that the number of research secretariat staff at each participating institution is insufficient to support the investigators. Therefore, to support the research secretariat in each participating center and to promote patient recruitment, clinical study manager and assistant clinical study manager in charge of the STAR-ACS conducted site visits. From Feburuary 2018 to July 2019, we visited 32 research institutions that did not register patients at that time. We held an explanatory meeting for doctors and clinical research coordinators (CRC) to accelerate patient enrollment.

A 20-min PowerPoint presentation was presented on the study outline and purpose, patient enrollment progress and research center ranking, introduction of activities to accelerate patient enrollment by the JCRTC, explanation of the inclusion/exclusion criteria, FAQ responses, and Q&A sessions.

### CRC Participation in STAR-ACS

The principal investigator, co-investigator, CRC, and research assistant collected data and performed the electronic data capture (EDC). Only individuals with EDC IDs can access the data entry system. The relationship between CRC participation and patient enrollment progress at the centers was evaluated using the CRC’s log-in history.

### Information Regarding Research Centers

We collected information on the participating centers, which included hospital type (university or community-based hospital), location (urban or rural), and number of hospital beds. Hospital information was obtained from the Residency Electronic Information System website (REIS, 2021) and each hospital’s official homepage. Hospitals in 20 cities designated by the Ministry of Internal Affairs and Communications and 23 wards in Tokyo were considered urban hospitals; the rest were considered rural hospitals.

### Statistical Analysis

Results are expressed as mean ± standard deviation or as numbers and proportions (%). Continuous and categorical variables were compared using the *t*-test and chi-squared test, respectively. Using multivariate logistic analyses, we assessed the association between patient enrollment and hospital information (including location, number of beds, and hospital category), site visit status, and CRC participation. Statistical analyses were performed using SAS version 9.4 (SAS Institute Inc., Cary, NC). *p*-values < 0.05 were considered statistically significant.

## Results

Table 1 shows the demographic data from 106 research centers. Of the 72 facilities with patient enrollment, 34 (47.2%) are located in urban areas; of the 34 facilities without patient enrollment, 23 (67.6%) are located in urban areas. Of the 72 facilities with patient enrollment, 29 (40.3%) are university hospitals; of the 34 facilities without patient enrollment, 14 (41.2%) are university hospitals. Of the 72 facilities with patient enrollment, CRC is active in 30 (41.7%); of the 34 facilities without patient enrollment, CRC is active in 4 (11.8%). Of the 72 facilities with patient enrollment, 28 (38.9%) perform site visits; of the 34 facilities without patient enrollment, 4 (11.8%) perform site visits. The average (±standard deviation (SD)) number of hospital beds is 664 ± 294 at the facilities with patient enrollment, while the average number of hospital beds is 580 ± 280(SD) at the facilities without patient enrollment. The following parameters differed in terms of patient enrollment and non-enrollment: urban area (47.2 vs. 67.6%), CRC participation (41.7 vs 11.8%), and site visit (38.9 vs 11.8%).

**TABLE 1:** Demographic characteristics (*n* = 106).

	Patient enrollment	Patient non-enrollment	*p*
Urban area	47.2% (34/72)	67.6% (23/34)	0.04^*^
University hospital	40.3% (29/72)	41.2% (14/34)	0.93
Number of hospital beds	664 ± 294	580 ± 280	0.17
CRC participation	41.7% (30/72)	11.8% (4/34)	<0.01^*^
Site visit	38.9% (28/72)	11.8% (4/34)	<0.01^*^

CRC = *clinical research coordinator.* :*means, p < 0.05.

Table 2 shows the multivariate logistic analysis results. The following factors were significantly associated with patient enrollment: urban area (odds ratio [OR] 0.33, 95% confidence interval [CI] 0.12–0.86, *p* = 0.02), CRC participation (OR 5.02, 95% CI 1.49–16.8, *p* < 0.01), and site visit (OR 4.54, 95% CI 1.31–15.7, *p* = 0.01).

**TABLE 2:** Factors associated patient enrollment (n = 106).

	Multivariate analysis		
	OR	95% CI	*P*
Urban area (vs. rural)	0.33	0.12–0.86	0.02^*^
University hospital (vs. community-based)	0.48	0.14–1.57	0.22
Number of hospital beds (per 100 units)	1.18	0.95–1.47	0.11
CRC participation	5.02	1.49–16.8	<0.01^*^
Site visit	4.54	1.31–15.7	0.01^*^

CRC = clinical research coordinator, OR = odds ratio, CI = confidence interval. ^*^means, p < 0.05.

**FIGURE 1:**
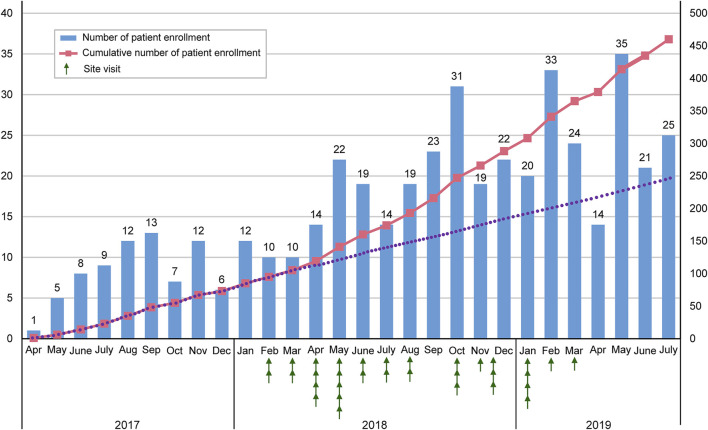
Transition in the number of enrolled patients.

Figure 1 depicts the transition in the number of enrolled patients from April 2017 to July 2019. The site visits started in Feburuary 2018. Thereafter, the number of enrolled patients increased.

## Discussion

### Summary of the Current Study

To the best of our knowledge, this is the first study to assess factors associated with accelerating patient enrollment in a clinical study using data from an ongoing multicenter prospective cohort study. Results showed that site visits and CRC participation had a significant effect on promoting patient enrollment. Rural hospitals were more effective in promoting patient enrollment than urban hospitals.

### Introduction of Previous studies

In a previous study, patient recruitment was an important factor for successful research (Prescott et al., 1999). However, delayed patient recruitment is frequently encountered in clinical research (Gates et al., 2004). Other studies have shown that associated factors include busy clinician schedules, staff shortages, research planning complexity, and memory lapses among researchers (Hetzel et al., 1998; Peto et al., 1993; Dickinson, 1994; Smyth et al., 1994;, 1998; Warlow, 2002; Prout et al., 2003). Failure to reach the target sample size compromises the research findings’ reliability and validity. Measures should therefore be taken to ensure sufficient participation.

### Advantage of Face-To-Face Communication

Compared with remote communication (e-mails, telephone calls), face-to-face communication during site visits was more effective, probably because it involves various types of visual non-verbal cues, auditory perception including intonation, and tactile information conveyed during body contact. Studies have reported the importance in other fields of communication through non-verbal information (Nishizaki et al., 2010; Steven and Julian, 1991; Waitzkin, 1984; Ishikawa et al., 2006; Roter et al., 2006). By visiting the study sites and meeting with researchers, the support staff’s enthusiasm and feelings regarding the study were communicated, which in turn facilitated patient enrollment.

The difference between the regular notifications (*via* e-mails, telephone calls, and newsletters) and site visits was the detailed explanation of the inclusion criteria. If selection criteria are directly stated upon visiting the centers, the researchers are more likely to determine whether the patients are eligible, will more likely be knowledgeable about STAR-ACS participation, and will seriously consider patient enrollment. Such site visits are therefore effective in promoting enrollment of candidates into clinical studies.

### Clinical Implication of CRC Participation

CRC involvement at the study sites was extremely effective in promoting patient enrollment. Collaboration between researchers and support staff is important for facilitating clinical research, given that the role of the CRC is particularly substantial (Pearl et al., 2003). Issues among physicians during clinical studies include schedule management, providing explanations to obtain informed consent, and preparing case report forms. CRC involvement reduces these burdens and increases research speed. CRCs are highly valued as staff liaisons and coordinators for other departments within and outside the sites and are essential for facilitating interprofessional collaborations. Studies have shown effective patient enrollment with full-time on-site CRCs (Shea et al., 1992).

### Differences Between Urban and Rural Areas

Rural hospitals are more effective in promoting patient enrollment than urban hospitals. The number of academic conferences in urban areas tend to be higher than that in rural areas. There is the possibility that hospital physicians in urban areas might spend more time attending or preparing for academic conferences compared to those in rural areas. Rural hospital physicians are more likely to concentrate on patient enrollment than urban physicians. There are significantly more urban hospital physicians than rural ones, and the former are more likely to focus on one organ/system. Patient visit information is therefore limited to their subspecialty. Rural hospital physicians are more likely to cover multiple disciplines. General physicians obtain more patient visit information (Nishizaki et al., 2020) and are therefore less likely overlook the enrollment of eligible candidates for clinical research.

### Limitations

The current study had four limitations. The first was the choice of sites visited. Selection bias regarding such sites cannot be eliminated because we emphasized the prompt promotion of patient enrollment and rapid completion of research. Thus, sites with a large number of patients with ACS were selected and visited. Second, sites with an EDC ID issued to the CRCs were associated with better CRC participation. However, the effect of CRC participation, which is affected by factors other than EDC entry, even in sites without an issued EDC ID, could not be ruled out. Third, we did not consider workload indicators, such as the number of patients and ambulances, in addition to the proportion of physicians. The results might therefore have been affected by the clinicians’ busy schedules. Forth, in general, CRC provides a wide range of research support for clinical researchers, including support for creating case report forms, assistance with explaining patient consent, management of patient visit dates and examination schedules according to the protocol, input of patient data to EDC, etc. In the present study, when the CRC only input patient data to the EDC, it was still counted as CRC participation. In fact, we did not obtain information about how much clinical research support CRC provided, other than patient data entry into the EDC.

We believe that an important factor in facilitating patient enrollment is balance between abundant support provided by clinical research supporters and the busyness of physicians who are in a position to recruit patients. In future studies, we hope to evaluate the relationship between this balance and the promotion of patient enrollment. In the current study, although we focused on observational studies, it will be necessary to verify whether the results can also be applied to interventional studies. As a next step, we would like to evaluate the factors that promote patient enrollment in multicenter clinical trials.

## Conclusion

In conclusion, site visits are effective in promoting patient enrollment in clinical research. Although electronic contact can facilitate research, site visits remain the most effective method for improving patient enrollment, particularly among those with rare diseases. On-site CRC participation is useful, and rural hospitals can achieve better patient enrollment than urban hospitals.

## Data Availability

The datasets in this study are not publicly available. Requests to access the datasets should be directed to the corresponding author.

## References

[B1] Campbell M. K. Snowdon C. Francis D. Elbourne D. McDonald A. M. Knight R. (2007). Recruitment to Randomised Trials: Strategies for Trial Enrollment and Participation Study. The STEPS Study. Health Technol. Assess. 11, iii–105. 10.3310/hta11480 17999843

[B4] Dickinson C. J. (1994). Clinical Research in the NHS Today. J. R. Coll. Physicians Lond. 28, 460–463. PubMed: 7807437. 7807437 PMC5401014

[B6] Gates S. Brocklehurst P. Campbell M. Elbourne D. (2004). Recruitment to Multicentre Trials. BJOG 111, 3–5. PubMed: 14687044. 10.1111/j.1471-0528.2004.00011.x 14687044

[B7] Hetzel M. R. Lee T. Prescott R. J. Woodhead M. Millar A. B. Peake M. (1998). Multi-centre Clinical Respiratory Research: a New Approach? J. R. Coll. Physicians Lond. 32, 412–416. PubMed: 9819730. 9819730 PMC9663106

[B8] Ishikawa H. Hashimoto H. Kinoshita M. Fujimori S. Shimizu T. Yano E. (2006). Evaluating Medical Students' Non-verbal Communication during the Objective Structured Clinical Examination. Med. Educ. 40, 1180–1187. PubMed: 17118111. 10.1111/j.1365-2929.2006.02628.x 17118111

[B10] Kitterman D. R. Cheng S. K. Dilts D. M. Orwoll E. S. (2011). The Prevalence and Economic Impact of Low-Enrolling Clinical Studies at an Academic Medical center. Acad. Med. 86, 1360–1366. PubMed: 21952064. 10.1097/ACM.0b013e3182306440 21952064 PMC3203249

[B12] Nishizaki Y. Shimizu T. Shinozaki T. Okubo T. Yamamoto Y. Konishi R. (2020). Impact of General Medicine Rotation Training on the In-Training Examination Scores of 11, 244 Japanese Resident Physicians: a Nationwide Multi-center Cross-Sectional Study. BMC Med. Educ. 20, 426. PubMed: 33187497. 10.1186/s12909-020-02334-8 33187497 PMC7666491

[B13] Nishizaki Y. Yoshioka Y. Hayano K. Miura J. Motomura K. Takei J. (2010). Medical Interview Skills and Patient Satisfaction Levels in a Setting Utilizing Electronic Medical Records. Gen. Med. 11, 17–23. 10.14442/general.11.17

[B14] Pearl A. Wright S. Gamble G. Doughty R. Sharpe N. (2003). Randomised Trials in General Practice-Aa New Zealand Experience in Recruitment. N. Z. Med. J. 116, U681. PubMed: 14657964. 14657964

[B15] Peto V. Coulter A. Bond A. (1993). Factors Affecting General Practitioners' Recruitment of Patients into a Prospective Study. Fam. Pract. 10, 207–211. PubMed: 8359613. 10.1093/fampra/10.2.207 8359613

[B16] Prescott R. J. Counsell C. E. Gillespie W. J. Grant A. M. Russell I. T. Kiauka S. (1999). Factors that Limit the Quality, Number and Progress of Randomised Controlled Trials. Health Technol. Assess. 3, 1–143. PubMed: 10683591. 10.3310/hta3200 10683591

[B17] Prout H. Butler C. Kinnersley P. Robling M. Hood K. Tudor-Jones R. (2003). A Qualitative Evaluation of Implementing a Randomized Controlled Trial in General Practice. Fam. Pract. 20, 675–681. PubMed: 14701891. 10.1093/fampra/cmg609 14701891

[B18] REIS (2021). Residency Electronic Information System. Available at: https://www.iradis.mhlw.go.jp/reis/common/ad0.xhtml (Accessed January 29, 2021).

[B19] Roter D. L. Frankel R. M. Hall J. A. Sluyter D. (2006). The Expression of Emotion through Nonverbal Behavior in Medical Visits. Mechanisms and Outcomes. J. Gen. Intern. Med. 21 (Suppl. 1), S28–S34. PubMed: 16405706. 10.1111/j.1525-1497.2006.00306.x PMC1484830 16405706

[B21] Shea S. Bigger J. T. Jr. Campion J. Fleiss J. L. Rolnitzky L. M. Schron E. (1992). Enrollment in Clinical Trials: Institutional Factors Affecting Enrollment in the Cardiac Arrhythmia Suppression Trial (CAST). Control. Clin. Trials 13, 466–486. PubMed: 1334819. 10.1016/0197-2456(92)90204-d 1334819

[B22] Smyth J. F. Mossman J. Hall R. Hepburn S. Pinkerton R. Richards M. (1994). Conducting Clinical Research in the New NHS: the Model of Cancer. United Kingdom Coordinating Committee on Cancer Research. BMJ 309, 457–461. PubMed: 7920132. 10.1136/bmj.309.6952.457 7920132 PMC2540931

[B23] Stein M. A. Shaffer M. Echo-Hawk A. Smith J. Stapleton A. Melvin A. (2015). Research START: a Multimethod Study of Barriers and Accelerators of Recruiting Research Participants. Clin. Transl. Sci. 8, 647–654. PubMed: 26643413. 10.1111/cts.12351 26643413 PMC4753776

[B24] Sully B. G. Julious S. A. Nicholl J. (2013). A Reinvestigation of Recruitment to Randomised, Controlled, Multicenter Trials: a Review of Trials Funded by Two UK Funding Agencies. Trials 14, 166. PubMed: 23758961. 10.1186/1745-6215-14-166 23758961 PMC3691846

[B25] Steven C. Julian B (1991). The Medical Interview: The Three-Function Approach. St. Louis, MO: MosbyYear Book, Inc. 225–238.

[B26] University Hospital medical information network (UMIN) (2017). A Prospective Observational Study of Bleeding and Thrombus Risk after Antithrombotic Treatment in Patients with Acute Coronary Syndrome with Atrial Fibrillation (Multicenter Study). Available at: https://upload.umin.ac.jp/cgi-open-bin/ctr/ctr_view.cgi?recptno=R000031122 (Accessed January 29, 2021).

[B27] Waitzkin H. (1984). Doctor-patient Communication. Clinical Implications of Social Scientific Research. JAMA 252, 2441–2446. PubMed: 6481931. 10.1001/jama.252.17.2441 6481931

[B28] Warlow C. (2002). Advanced Issues in the Design and Conduct of Randomized Clinical Trials: the Bigger the Better? Stat. Med. 21, 2797–2805. PubMed: 12325095. 10.1002/sim.1283 12325095

